# Positively Valenced Stimuli Facilitate Creative Novel Metaphoric Processes by Enhancing Medial Prefrontal Cortical Activation

**DOI:** 10.3389/fpsyg.2013.00211

**Published:** 2013-04-26

**Authors:** Karuna Subramaniam, Mark Beeman, Miriam Faust, Nira Mashal

**Affiliations:** ^1^Department of Psychiatry, University of California San FranciscoSan Francisco, CA, USA; ^2^Department of Psychology, Northwestern UniversityEvanston, IL, USA; ^3^Multidisciplinary Brain Research Center, Bar-Ilan UniversityRamat-Gan, Israel; ^4^Department of Psychology, Bar-Ilan UniversityRamat-Gan, Israel; ^5^School of Education, Bar-Ilan UniversityRamat-Gan, Israel

**Keywords:** novel metaphors, conventional metaphors, affective valence, medial prefrontal cortex, fMRI, creativity

## Abstract

A metaphor is a figure of speech in which a subject is symbolic of another unrelated object. In the present study, we examined neural patterns associated with both novel unfamiliar and conventional familiar metaphoric processing, and how these patterns are modulated by affective valence. Prior to fMRI scanning, participants received a list of word pairs (novel unfamiliar metaphors as well as conventional familiar metaphors) and were asked to denote the valence (positive, negative, or neutral) of each word pair. During scanning, participants had to decide whether the word pairs formed meaningful or meaningless expressions. Results indicate that participants were faster and more accurate at deciding that positively valenced metaphors were meaningful compared to neutral metaphors. These behavioral findings were accompanied by increased activation in the medial prefrontal cortex (mPFC), posterior cingulate cortex (PCC), and the right inferior parietal lobe (RIPL). Specifically, positively valenced novel unfamiliar metaphors elicited activation in these brain regions in addition to the left superior temporal gyrus when compared to neutral novel metaphors. We also found that the mPFC and PCC mediated the processing of positively valenced metaphors when compared to negatively valenced metaphors. Positively valenced conventional metaphors, however, elicited different neural signatures when contrasted with either neutral or negatively valenced conventional metaphors. Together, our results indicate that positively valenced stimuli facilitate creative metaphoric processes (specifically novel metaphoric processes) by mediating attention and cognitive control processes required for the access, integration, and selection of semantic associations via modulation of the mPFC. The present study is important for the development of neural accounts of emotion-cognition interactions required for creativity, language, and successful social functioning in general.

## Introduction

A metaphor is a figure of speech that describes a subject by stating that it is comparable to another unrelated object (i.e., *Her eyes were glistening jewels*). Metaphors are pervasive in everyday life; they are universal and ubiquitous across cultures and are essential for successful communication and social interactions (Gibbs, 1994). Metaphoric comprehension is considered a type of creative cognition which involves at least two processing stages, unlike literal utterances which involve only one stage (Giora, 2007). According to the standard pragmatic model (Grice, 1975), when trying to derive meaning from metaphors, the literal interpretation is first computed and rejected as inappropriate, and is then replaced with a non-literal figurative meaning. Thus, metaphoric comprehension involves distinct higher-order processes of understanding and experiencing one thing in terms of another (i.e., in the example above, eyes are experienced as jewels).

An alternative metaphoric model, the sequential metaphoric model of Grice (1975) and Glucksberg (2003), suggests that during metaphoric comprehension, the literal meaning does not have unconditional priority over the figurative metaphoric interpretation and that the figurative meanings can be computed in the same manner as literal meanings. In other words, metaphors (e.g., *that fighter is a lion*) are taken literally as categorical assertions by inclusion of the topic (i.e., fighter) as a member of a superordinate category exemplified by the vehicle (lion). A different approach highlights the similarity between metaphors and analogies, both of which are based on structural mapping (Gentner, 1983). Gentner’s (1983) structural mapping model assumes that the common properties of the topic and the vehicle terms are identified and extracted and are then exhaustively checked against one another so that the properties that “match” can serve as the foundation for the metaphor.

According to a third model, the Gradient Salience Hypothesis (Giora, 1997, 2003), metaphoric processing is modulated by meaning salience, which is determined by the conventionality, frequency, and familiarity of the words. Taking all these models into account, in our prior study, we examined whether metaphoric comprehension is computed in a similar manner to literal meanings or whether metaphoric comprehension involves more complex, higher-order processing (Subramaniam et al., 2012). Specifically, we assessed processes related to conventional (familiar) metaphors (e.g., *brain freeze*) as well as novel (unfamiliar) metaphors (e.g., *unfenced idea*) (Fraser, 1998). Conventional metaphoric processing involves recalling a familiar closely connected meaning (Amanzio et al., 2008). By contrast, novel metaphoric processing is a type of creative cognition that involves formulating new meanings from unfamiliar expressions that have not been previously encountered (Subramaniam et al., 2012). In the case of conventional metaphors in which the metaphorical meaning represents a familiar salient meaning (Gibbs, 1980; Giora and Fein, 1999a), it is the figurative meaning that is processed first, without having accessed the less salient (literal) meaning (Gibbs, 1980; Giora and Fein, 1999b). On the other hand, unfamiliar metaphorical meanings during novel metaphoric comprehension are not lexicalized; hence these meanings are non-salient and are only accessed after the more salient (literal) meanings have been retrieved. Thus, processing novel metaphors tend to involve greater attention and cognitive control processes (Grice, 1975; Chiappe and Chiappe, 2007) such that participants need to recognize distant and unfamiliar associations between words; to eliminate interpretations that are meaningless in order to converge upon selecting a coherent meaningful interpretation.

Additionally, prior research has also revealed that comprehension of creative metaphoric expressions is associated with efficient working memory and cognitive control. For example, Chiappe and Chiappe (2007) examined the association between the time required to generate metaphoric interpretations with both working memory capacity and inhibitory control. The results showed that working memory capacity and inhibitory control predicted both the time required to interpret metaphors and the quality of those interpretations. Furthermore, a recent study showed that comprehension of both unfamiliar and familiar metaphors correlated with working memory capacity and, in particular, with backward digit span working memory processes (Mashal, in press). Finally, in our previous paper we revealed that creative solving required greater attentional and cognitive computations, which was facilitated by a positive mood and was mediated by the medial prefrontal cortex/anterior cingulate cortex (mPFC/ACC) (Subramaniam et al., 2009). Extending upon these prior findings, in the present study, we predicted that positively valenced stimuli would also enhance creative metaphoric-solving processes, via similar neural mechanisms as a positive mood, by regulating attention and cognitive control mechanisms within the mPFC/ACC.

In our previous metaphoric fMRI study, we used the repetition suppression paradigm for the first time to investigate the neural patterns underlying conventional and novel metaphorical processes and, in particular, we examined how novel metaphors became conventionalized in the brain (Subramaniam et al., 2012). Repetition suppression refers to brain regions that show reduced neural activity for repeated stimuli presentations. This reduced activation indicates a facilitation of retrieving the same familiar information during the second repeated exposure to these stimuli when compared to the first. Repetition enhancement, on the other hand, refers to brain regions that show increased activation during the second repeated exposure to stimuli when compared to the first, indicating the formulation of new meaning in the mental lexicon (Subramaniam et al., 2012).

In this previous metaphoric fMRI study (Subramaniam et al., 2012), prior to scanning, participants read half of the novel metaphoric expressions and half of the conventional metaphoric expressions that they would later view again in the scanner. When novel metaphors were viewed a second time, we found repetition enhancement effects (increased activation) in several regions mediating the formulation of meaning, which included: bilateral inferior parietal gyri, precuneus/posterior cingulate cortex (PCC), and mPFC. However, no brain areas showed repetition suppression effects during the repeated exposure to novel metaphors. The lack of repetition suppression indicates that meaning had not yet been formulated during the first exposure in order for the retrieval of this meaning to be facilitated at the second exposure. As expected, repeated exposure to conventional metaphors elicited repetition suppression (reduced activation) within the left superior temporal gyrus (LSTG)/supramarginal gyrus, indicating facilitation of familiar meaning retrieval from the mental lexicon. These findings indicate that novel and conventional metaphorical comprehension involve different neural regions and processes.

We found that during novel metaphoric comprehension, regions such as the mPFC, PCC, and bilateral inferior parietal/temporal (IPL) cortices support meaning conceptualization and storage of novel semantic relations (Subramaniam et al., 2012). We now extend upon these prior findings in the present study. Here, we use fMRI to investigate not just the neural processes mediating metaphoric processing, but how different affective processes can modulate metaphoric comprehension in distinct ways. In other words, we examine whether any affective valence-modulated changes in neural signal differ between two types of metaphorical processes (novel unfamiliar metaphors and conventional familiar metaphors). Specifically, we hypothesized that stimuli classified as “positive” would enhance creative novel metaphoric processes by enhancing activation within the regions that revealed repetition enhancement effects during meaning formulation (mPFC, PCC, and bilateral IPL).

We know from prior research that a positive mood has been shown to facilitate creative problem solving across a broad range of settings (Isen et al., 1987b; Estrada et al., 1994; Ashby et al., 1999; Isen, 1999; Rowe et al., 2007). In particular, the mPFC/ACC is known to regulate attention and cognitive control processes in the frontal cortex (Bush et al., 2000; Botvinick et al., 2004; Kerns et al., 2004), and is specifically involved in the cognitive regulation of positive mood and positive stimuli (Subramaniam et al., 2009; Sass et al., 2012). We, therefore, predicted that positively valenced stimuli are likely to facilitate creative novel metaphoric comprehension by enhancing activity within the mPFC/ACC to enhance the detection of competing associations during novel metaphoric meaning formulation. We also know that the PCC mediates visuospatial attention processes (Small et al., 2003) and that positive mood states and positively valenced stimuli promote a broader scope of attention (Gasper and Clore, 2002; Fenske and Eastwood, 2003; Srinivasan and Hanif, 2010). Thus, it is also possible that positively valenced stimuli may enhance visual attention processes in the PCC during novel metaphoric comprehension.

Prior research indicates that the right hemisphere (RH) is activated when participants process distantly related meanings and performs more “coarse semantic coding” (Jung-Beeman, 2005). Converging evidence from brain damaged patients (Brownell et al., 1984), behavioral divided visual field (DVF) studies (Faust and Mashal, 2007; Mashal and Faust, 2009), Transcranial Magnetic Stimulation (TMS) (Pobric et al., 2008), and fMRI studies (Mashal et al., 2005, 2007) all attribute a special role to the RH during the processing of novel metaphors. Therefore, another putative explanation as to how positively valenced stimuli may facilitate meaningful formulation from non-salient metaphors is through enhancing integration of distantly related associations via right temporal/parietal activation. Finally, novel metaphoric comprehension also requires the left hemisphere (LH) to perform more fine semantic coding (Jung-Beeman, 2005) during the integration and selection of more closely connected meanings from the non-salient metaphors (Geschwind, 1965; Binder et al., 2009; Bambini et al., 2011).

Taken together, we hypothesized that positively valenced stimuli may modulate some, or all, of the regions that show repetition enhancement effects during novel metaphoric meaning formulation. This may be done initially via modulation of the right temporal/parietal cortex during more coarse semantic coding required for the integration of distant word pair associations. Next, mPFC/PCC modulation would be required in order to regulate attention and cognitive control processes. Increased control exerted by the mPFC would likely facilitate fine semantic coding supported by the left temporal/parietal cortex required for the formulation of closely connected more salient meanings.

It must be noted that we are not stating that positive mood and positively valenced stimuli will always unconditionally enhance creative performance. Rather, consistent with Kaufmann and Vosburg (1997) findings, we find that mood effects on creativity are task-specific. Indeed, prior studies have shown that while a positive mood can broaden attention on creative tasks, it can also impair selective attention, leading to increased distractibility (Dreisbach and Goschke, 2004; Rowe et al., 2007). In the present study, we predicted that the effects of positive mood and positively valenced stimuli can impair or enhance cognition depending on whether task performance is enhanced by a broadening of attention, or impaired by a global scope of attention. Specifically, we hypothesized that when participants viewed novel unfamiliar metaphors, they would need to have broader attention/increased semantic access to detect multiple possible word pair interpretations, which would be facilitated by positively valenced stimuli. Additionally, in the present study, participants had a very short time to decide the meaningfulness of each word pair (maximum time = 3.5 s). Thus, the current predictions are also consistent with the Kaufmann and Vosburg (1997) findings that positive moods and stimuli enhance creativity for tasks that fulfill a satisficing criteria (i.e., tasks which have limited response times facilitating cognitive satisfaction rather than cognitive elaboration and optimization).

Taking into account the evidence from prior findings, we predicted that positively valenced stimuli would enhance signal within the regions that showed repetition enhancement effects for novel metaphoric processes (i.e., mPFC, PCC, left, and right temporal/parietal regions). No studies to date have been conducted on how negatively valenced stimuli may modulate metaphoric processes. As such, in order to generate novel findings and to minimize Type II error and miss true signal in regions other than those consistent with our *a priori* hypothesis, we also conducted the below exploratory whole-brain analyses. The whole-brain analyses enabled examination of how different types of affective valence could modulate neural activity associated with metaphoric processes in common and dissociable ways. The whole-brain analyses also allowed investigation throughout the brain, of whether any valence-modulated changes in neural signal differed between the two types of metaphors (novel versus conventional). Our goals were:

To identify the brain regions that are modulated by positively valenced stimuli in general (both conventional and novel metaphors) when compared to neutral and negatively valenced stimuli, as well as to identify brain regions that are modulated by negatively valenced stimuli when compared to neutral stimuli.
To identify all brain areas that demonstrate signal change for positively valenced novel metaphors versus neutral novel metaphors (PosNM versus NeutNM), as well as for positively valenced conventional metaphors versus neutral conventional metaphors (PosCM versus NeutCM).
To identify all brain areas that demonstrate signal change for negatively valenced novel metaphors versus neutral novel metaphors (NegNM versus NeutNM), as well as for negatively valenced conventional metaphors versus neutral conventional metaphors (NegCM versus NeutCM).
To identify all brain areas that demonstrate signal change for positively valenced novel metaphors versus negatively valenced novel metaphors (PosNM versus NegNM), as well as for positively valenced conventional metaphors versus negatively valenced conventional metaphors (PosCM versus NegCM).

## Materials and Methods

### Participants and procedure

Fourteen undergraduate subjects (mean age = 25.5, SD = 8.16, seven males), participated in the study. All 14 participants were neurologically healthy, right-handed, and native speakers of English. After giving informed consent, 14 participants completed an emotional valence questionnaire outside the scanner, and then performed a semantic judgment task in the scanner. Data from one participant was excluded from the analysis due to poor fMRI signal.

The same fMRI scanning procedure was used here as in our previous study (Subramaniam et al., 2012); however, the study objectives, fMRI analyses, and results are entirely different. The present study is a follow-up metaphorical study in which we examine how different affective processes can modulate metaphoric comprehension in distinct ways, and is informed from the findings of our previous paper (Subramaniam et al., 2012). In this prior study, we revealed different and distinct neural mechanisms underlying conventional and novel metaphors when viewed for the first time. We also revealed how novel metaphors seen for the first time had been processed like unrelated words but when viewed again became conventionalized in the brain, revealing similar neural patterns to conventional metaphors (Subramaniam et al., 2012).

### Stimuli

Stimuli included 136 two-word English metaphoric expressions, and 34 unrelated word pairs (see Appendix for examples). The two words formed one of three types of semantic relations (conditions): conventional metaphoric (*heated debate*), novel metaphoric (*caged cry*), or unrelated (*devastated snow*) word pairs. Two pretests were performed in order to determine the degree of plausibility and the degree of familiarity of each metaphoric expression. The aim of the first pretest was to determine the type of each two-word expression (metaphoric or unrelated). Twelve judges (who did not participate in the experiment) were presented with a list of two-word expressions and were asked to decide whether each expression was metaphorically plausible or not plausible. Expressions that were rated by at least 75% of the judges as either metaphorically plausible or not plausible were selected as metaphoric expressions or unrelated word pairs, respectively.

In order to distinguish between novel metaphors and conventional metaphors, another group of 10 judges were presented with a list of only the plausible metaphors from the first pretest. They were asked to rate their degree of familiarity on a five-point familiarity scale ranging from 1 (highly non-familiar) to 5 (highly familiar). Metaphoric expressions which were scored lower than 3.25 on the familiarity scale were selected as novel metaphors (rating average 2.73), whereas those that were scored higher than four on this scale were selected as conventional metaphors (rating average 4.84) for the present study. The novel metaphoric expressions were rated as significantly less familiar than the conventional ones [*t*(67) = 41.15, *p* < 0.0001].

Stimuli were then balanced between conditions according to word frequency. The novel metaphors, conventional metaphors, and the unrelated word pairs did not differ in terms of average word frequency per million, *F* < 1 (Kucera and Francis, 1967). In addition, stimuli were balanced between conditions according to syntactic form. The second word of each expression was always a noun, whereas the first word was either a noun or adjective. Grammatical category was counterbalanced across the four types of word pairs. Thus, each condition contained equal numbers of nouns and adjectives. Complete methodological details on metaphoric ratings of frequency and familiarity are also described in our previous paper (Subramaniam et al., 2012).

### Metaphor paradigm

Prior to fMRI scanning, participants received a list of word pairs which consisted of novel unfamiliar metaphors and conventional familiar metaphors that they would later view again in the scanner. Participants were instructed: “the following expressions are either familiar (e.g., *shady character*) or unfamiliar (e.g., *solitude shards*) metaphors. Please denote the valence of each of the following expressions.” Participants had to mark an X in the column they selected for word pairs with positive connotations, negative connotations, or neutral connotations. For example, conventional metaphors such as “beautiful mind,” “visual field,” and “sour grapes” were typically classified as having positive, neutral, and negative connotations, respectively. Similarly, novel metaphors such as “joy bits,” “memory phantoms,” and “caged cry” were also typically classified as having positive, neutral, and negative connotations.

We used fMRI to measure brain activity associated with each participant’s individual subjective ratings of the six different types of meaningful metaphors: positive novel metaphors, negative novel metaphors, neutral novel metaphors, positive conventional metaphors, negative conventional metaphors, and neutral conventional metaphors. During scanning, participants were presented with word pairs and had to decide whether the word pairs formed meaningful or meaningless expressions. Participants were informed that some of the word pairs were metaphorical expressions, which had a figurative meaning beyond the literal meaning of the individual words whereas other word pairs were completely devoid of any meaning (unrelated words). Participants were given 34 novel metaphors, 34 conventional metaphors, and 34 unrelated word pairs in the scanner. For conventional metaphors, such as “lion heart” and “steel convictions,” participants understood and processed these expressions rapidly, similar to literal expressions. By contrast, for novel metaphors such as “unfenced idea” and “joy bits,” participants needed to engage additional cognitive processes to form meanings for these novel unconventional word pairs, compared to conventional metaphorical expressions. Unrelated pairs included words such as “cheek brains” which were completely devoid of meaning. fMRI signal corresponding to unrelated word pair processing is reported in our prior paper (Subramaniam et al., 2012) but not in the present study because participants did not pre-classify unrelated words into positive, negative, or neutral categories prior to scanning. Therefore, the objective of the present study was to examine affective valence-modulated changes during meaningful conventional and novel metaphoric processes rather than during unrelated words.

The session began with a fixation cross that remained on the screen for 10 s. Each trial in the scanner began with a word pair (i.e., “ebbing fame”) that was presented on the screen for 2100 ms followed by a blank screen of 1400 ms duration. Participants were instructed to decide as quickly as possible, whether the expression was meaningful (i.e., left button-press with their right hand) or meaningless (i.e., right button-press with their right hand) as soon as they read each expression. Participants were given a limited time window (3500 ms) to respond. Thirty four additional fixation points (each presented for 3500 ms) were randomly interspersed between trials in order to jitter the events and optimize deconvolution of the fMRI signal from successive events.

### Image acquisition

Fourteen fMRI participants performed the metaphorical task during scanning, which for all participants occurred in the same Siemens Trio (3 T) scanner and eight channel head coil, with the same scanning protocol, at Northwestern’s Center for Advanced MRI. Head motion was restricted with plastic calipers built into the coil and a vacuum pillow. The functional imaging sequence was optimized for detection of the BOLD effect (Ogawa et al., 1992) including local shimming and 10 s of scanning prior to data collection to allow the MR signal to reach equilibrium. Functional imaging used a gradient echo echo-planar sequence (TR = 2 s for 38 3-mm slices, TE = 20 ms, matrix size 64 × 64 in 220-mm field of view). Each functional scan was synchronized with the onset of the first trial. Anatomical high-resolution images were acquired in the same plane, with T1-weighted images parallel to the ACPC plane.

### Image analyses

Images were analyzed using Matlab (Mathworks Inc.) and SPM2 software (www.fil.ion.ucl.ac.uk/spm). Images were realigned to correct for motion artifacts using a six-parameter rigid body affine transformation. The resulting images were normalized to a standard stereotaxic space [Montreal Neurological Institute (MNI) Template] using a 12 parameter affine/non-linear transformation and spatially smoothed with a 10-mm full-width half maximum isotropic Gaussian kernel. Data were submitted to a whole-brain General Linear Model analysis, fitting a reference canonical hemodynamic response function to each event. Image intensity was scaled to the mean global intensity of each time series.

Whole-brain contrasts of interest were performed on individual subject data from correct trials. For each participant, we compared activity during onsets of each of the six metaphorical conditions that were correctly classified as meaningful: positive novel metaphors, negative novel metaphors, neutral novel metaphors, positive conventional metaphors, negative conventional metaphors, and neutral conventional metaphors. Second-level random-effect one-sample *t*-tests were then conducted for all participants in the group. Reported results are significant at a threshold of combining *t* values (at an alpha *p* value of 0.001 uncorrected for multiple comparisons), and cluster size (at least 50 voxels or 400 mm^3^ in volume) in which each voxel showed reliable signal change across all participants for the specific contrast of interest (see Table 1). Consistent with our *a priori* hypothesis, whole-brain analysis of positively valenced versus neutral novel metaphoric comprehension revealed the same four brain regions [i.e., mPFC, PCC, LIPL, and right inferior parietal lobe (RIPL)] which also revealed repetition enhancement for novel metaphoric processing in our previous paper (Subramaniam et al., 2012). For subsequent analyses, we extracted mean beta signal for each valence (positive, neutral, and negative) as well as for each metaphoric condition (conventional and novel) from the following region of interest (ROI) centroids reported in Subramaniam et al. (2012): mPFC (14, 38, −12), PCC (−14, −36, 42), LIPL (−42, 8, −26), and RIPL (48, −38, 42). Mean beta signals for each ROI were then entered into a 2 × 3 repeated-measures ANOVA in Statistica in order to examine interaction effects in fMRI signal between metaphor type × valence.

**Table 1:** **Whole-brain contrasts showing clusters of activation at a threshold of *p* < 0.001 uncorrected, and with cluster size of at least 50 voxels or 400 mm^3^ in volume**.

Region	BA	*T*-max value	Cluster size (voxels)	Cluster size (mm^3^)	*X* *Y* *Z* (mm)
**A. POS.METAPHORS > NEUT.METAPHORS**					
mPFC/ACC	25, 32	7.91	374	2992	12, 30, −16
RIPL	40	5.07	192	1536	46, −46, 40
PCC	31	5.5	58	464	−14, −42, 38
**B. NEG.METAPHORS > NEUT.METAPHORS**					
RIPL	40	5.24	62	496	32, −42, 42
RMFG	11	6.45	57	456	36, 40, −16
**C. POS.METAPHORS > NEG.METAPHORS**					
Cingulate gyrus	24, 31	8.36	167	1336	−20, −10, 44
mPFC/RSFG	10	4.97	51	408	24, 58, 14
**D. POS.NM > NEUT.NM**					
mPFC/ACC	10, 25, 32	7.52	437	3496	14, 38, −12
PCC	31	5.05	108	864	−14, −36, 42
LSTG	21, 38	6.35	81	648	−42, 8, −26
RIPL	40	5.21	74	592	48, −38, 42
**E. POS.CM > NEUT.CM**					
R. Putamen/insula	13	8.81	102	816	34, −18, −4
RIPL	40	4.98	76	608	52, −44, 32
**F. NEUT.CM > POS.CM**					
LSFG	8	5.17	91	728	−6, 36, 54
LMTG	21	4.87	63	504	−64, −38, −6
**G. NEG.NM > NEUT.NM**					
RIPL	40	5.82	113	904	56, −32, 52
**H. NEG.CM > NEUT.CM**					
L. Inf. Occipital Gyrus	18	9.05	330	2640	−34, −88, −6
RSPL	7	5.51	119	952	22, −68, 62
**I. NEUT.CM > NEG.CM**					
LSFG	8	5.63	302	2416	−16, 30, 52
**J. POS.NM > NEG.NM**					
RPHC	36	5.33	51	408	18, −34, −14
mPFC/ACC	10	6.58	50	400	16, 44, −10
RMFG	9	5.29	50	400	30, 10, 42
**K. NEG.NM > POS.NM**					
L. Inf. Occipital Gyrus	18	6.06	56	448	−30, −82, −12
**L. NEG.CM > POS.CM**					
LFFG	37	5.26	55	440	−46, −64 −22

*mPFC/ACC, medial prefrontal cortex/anterior cingulate cortex; RIPL, right inferior parietal lobe; PCC, posterior cingulate cortex; RMFG, right middle frontal gyrus; LSFG, left superior frontal gyrus; LMTG, left middle temporal gyrus; RSPL, right superior parietal lobe; RPHC, right parahippocampal cortex; LFFG, left fusiform gyrus*.

## Results

### Behavioral results

#### Reaction times

**Figure 1:**
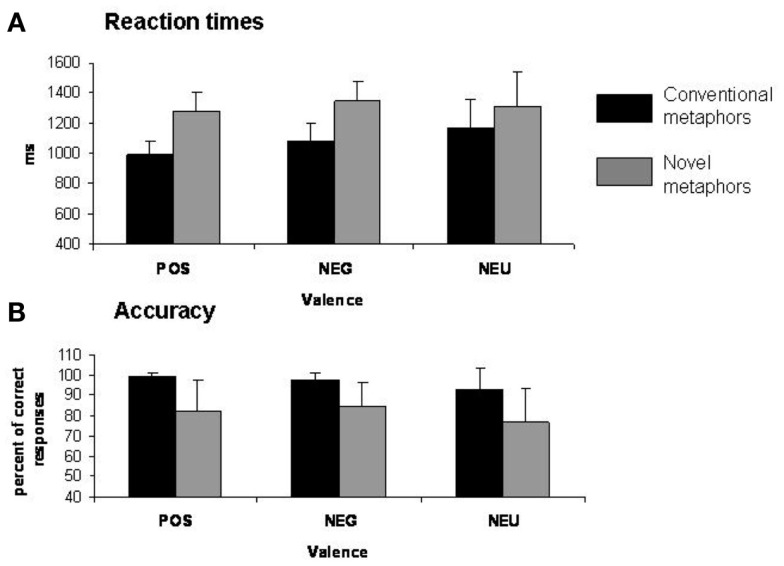
**Behavioral results**. **(A)** Mean reaction times (SE) for each condition. **(B)** Percent of correct responses (SE) for each condition.

Participants were asked to decide if each stimulus represented a meaningful or meaningless expression in the scanner. Reaction times (RT) were collected during scanning on accurate trials for each of the six types of stimuli: positive novel metaphors, negative novel metaphors, neutral novel metaphors, positive conventional metaphors, negative conventional metaphors, and neutral conventional metaphors. A 2 × 3 repeated-measures ANOVA with type of metaphor (conventional, novel) and affective valence (positive, negative, neutral) for RT of meaningful responses revealed a main effect of stimulus type [*F*(1, 12) = 31.60, *p* = 0.0001] (Figure 1A). Tukey *post hoc* comparisons revealed that participants made faster (meaningful) judgments for conventional metaphors (Mean = 1082 ms, SD = 150) when compared to novel metaphors (Mean = 1312 ms, SD = 169) (*p* = 0.0003). Although there was no significant (metaphor type × valence) interaction (*p* = 0.20), we did find a main effect of valence [*F*(2, 24) = 3.51, *p* = 0.046]. The main effect of valence was driven by participants making faster meaningful judgments for positively valenced metaphors (Mean = 1137 ms, SD = 180) when compared to neutral metaphors (Mean = 1238 ms, SD = 229) (*p* = 0.048). No significant difference in RT was found between positively valenced metaphors when compared to negatively valenced metaphors (Mean = 1217 ms, SD = 176) (*p* = 0.13) or between negatively valenced metaphors and neutral metaphors (*p* = 0.76).

#### Accuracy

A 2 × 3 repeated-measures ANOVA with type of metaphor (conventional, novel) and affective valence (positive, negative, neutral) for accuracy was calculated for trials correctly classified as meaningful in the scanner. The main effect of stimulus type was significant [*F*(1, 12) = 15.01, *p* = 0.002] (Figure 1B). Tukey *post hoc* comparisons revealed that participants correctly made more meaningful judgments for conventional metaphors (Mean = 96.68%, SD = 6.62) when compared to novel metaphors (Mean = 81.34%, SD = 15.05) (*p* = 0.002). Interestingly, although there was no metaphor type × valence interaction (*p* = 0.54), we found a significant main effect of valence [*F*(2, 24) = 5.40, *p* = 0.012]. Furthermore, Tukey *post hoc* comparisons revealed that participants made more meaningful judgments for positively valenced stimuli (Mean = 90.82%, SD = 14.07) when compared to neutral stimuli (Mean = 84.89%, SD = 16.17) (*p* = 0.031), and also made more meaningful judgments (Mean = 91.33%, SD = 10.41) for negatively valenced stimuli when compared to neutral stimuli (*p* = 0.018). No significant difference in accuracy was found between positively valenced metaphors when compared to negatively valenced metaphors (*p* = 0.97).

### fMRI results

#### ROI analyses

**Figure 2:**
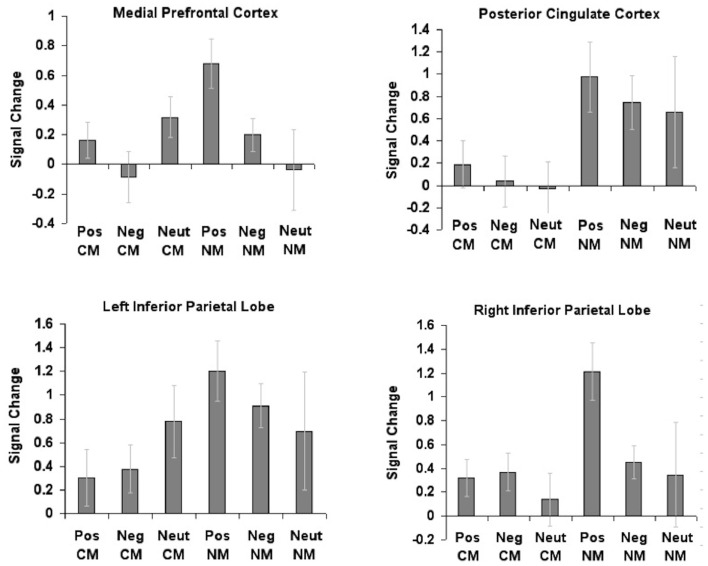
**Region of interest (ROI) analyses conducted for the four regions showing repetition enhancement effects from the activation centroids, previously described in Subramaniam et al. (2012)**. Mean beta signal extracted within each region for each metaphor condition (conventional, novel) and valence (positive, neutral, negative) revealed significant interaction effects between metaphor type by valence in only one region, the medial prefrontal cortex.

In accordance with our *a priori* hypothesis, we conducted 2 × 3 repeated-measures ANOVAs with metaphor type (conventional, novel) and valence (positive, negative, neutral) as within subject factors within the four regions (mPFC, RIPL, LIPL, and PCC) that showed significant repetition enhancement effects for metaphoric processes, as described in Subramaniam et al. (2012) (Figure 2).

Results revealed a marginally significant main effect of valence in mPFC *F*(2, 24) = 2.80, *p* = 0.081. Planned comparisons indicated increased mPFC activation for positively valenced metaphors when compared to negatively valenced metaphors (*p* = 0.033). We also found that the two-way stimulus type × valence interaction was significant, *F*(2, 24) = 3.65, *p* = 0.041. Planned comparisons revealed greater mPFC activation for positively valenced novel metaphors when compared to neutral novel metaphors (*p* = 0.006), and marginally greater mPFC activation when compared to negatively valenced novel metaphors (*p* = 0.054). The main effect of stimulus type was not significant (*p* = 0.35).

Within PCC, we found a main effect of stimulus type [*F*(1, 12) = 14.07, *p* = 0.002]. Planned comparisons indicated increased PCC activation for novel metaphors when compared to conventional metaphors (*p* = 0.002). However, neither the main effect of stimulus type nor the two-way interaction was significant (all *p* > 0.05).

The two-way repeated-measures ANOVA in LIPL showed a marginally significant main effect of stimulus type [*F*(1, 12) = 4.26, *p* = 0.061]. Planned comparisons revealed marginally greater LIPL activation for novel metaphors when compared to conventional metaphors (*p* = 0.061). However, neither the main effect of valence nor the two-way interaction was significant (all *p* > 0.05).

The two-way repeated-measures ANOVA in RIPL showed a marginally significant main effect of stimulus type [*F*(1, 12) = 4.55, *p* = 0.054]. Planned comparisons revealed marginally greater activation within RIPL for novel metaphors when compared to conventional metaphors (*p* = 0.054). The main effect of valence was significant [*F*(2, 24) = 3.41, *p* = 0.050], indicating greater RIPL activation for positively valenced metaphors when compared to neutral metaphors (*p* = 0.017). The two-way interaction was not significant (*p* > 0.05).

#### Exploratory whole-brain analyses

In order to minimize Type II error and miss true signal in regions other than those consistent with our *a priori* hypothesis, we conducted the whole-brain analyses described below. We investigated whether different types of affective valence-modulated neural activity associated with metaphoric processes in distinct ways. We also examined whether any valence-modulated changes in neural signal differed between the two types of metaphors (novel versus conventional).

### Does valence modulate metaphoric processing in the brain?

**Figure 3:**
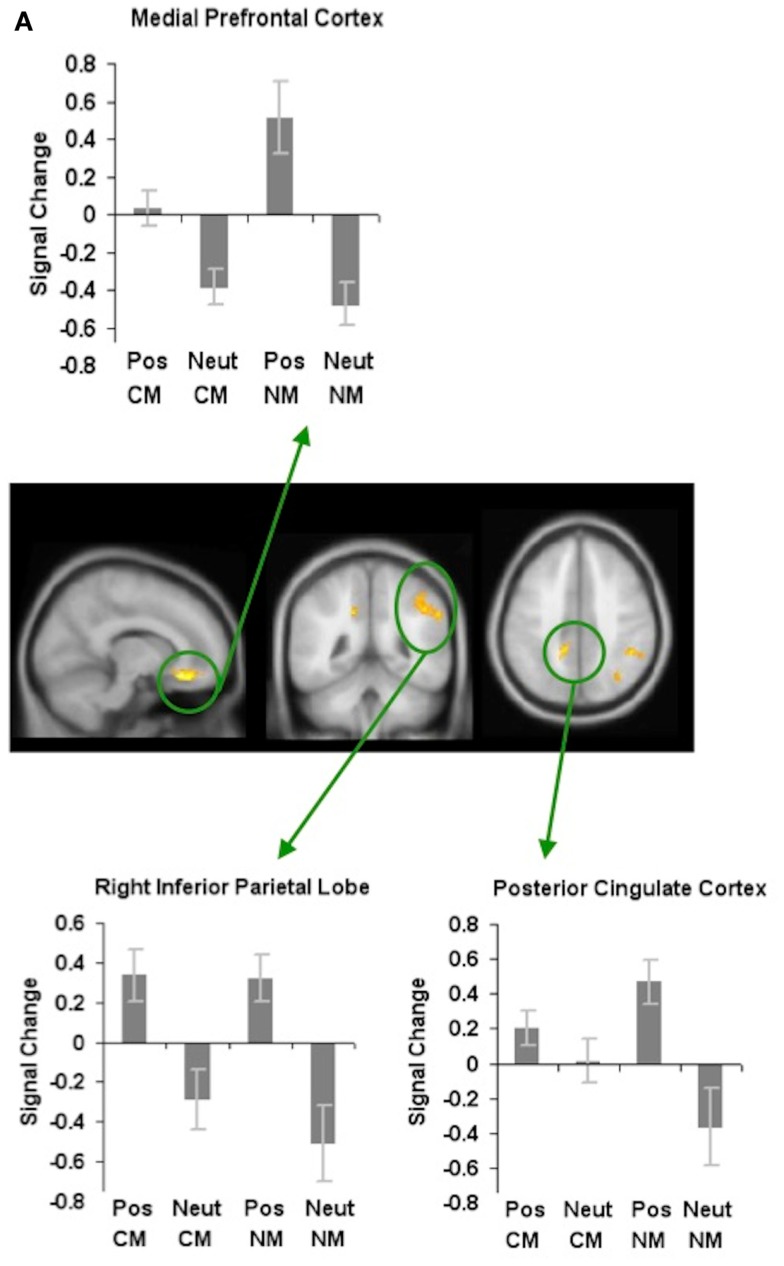

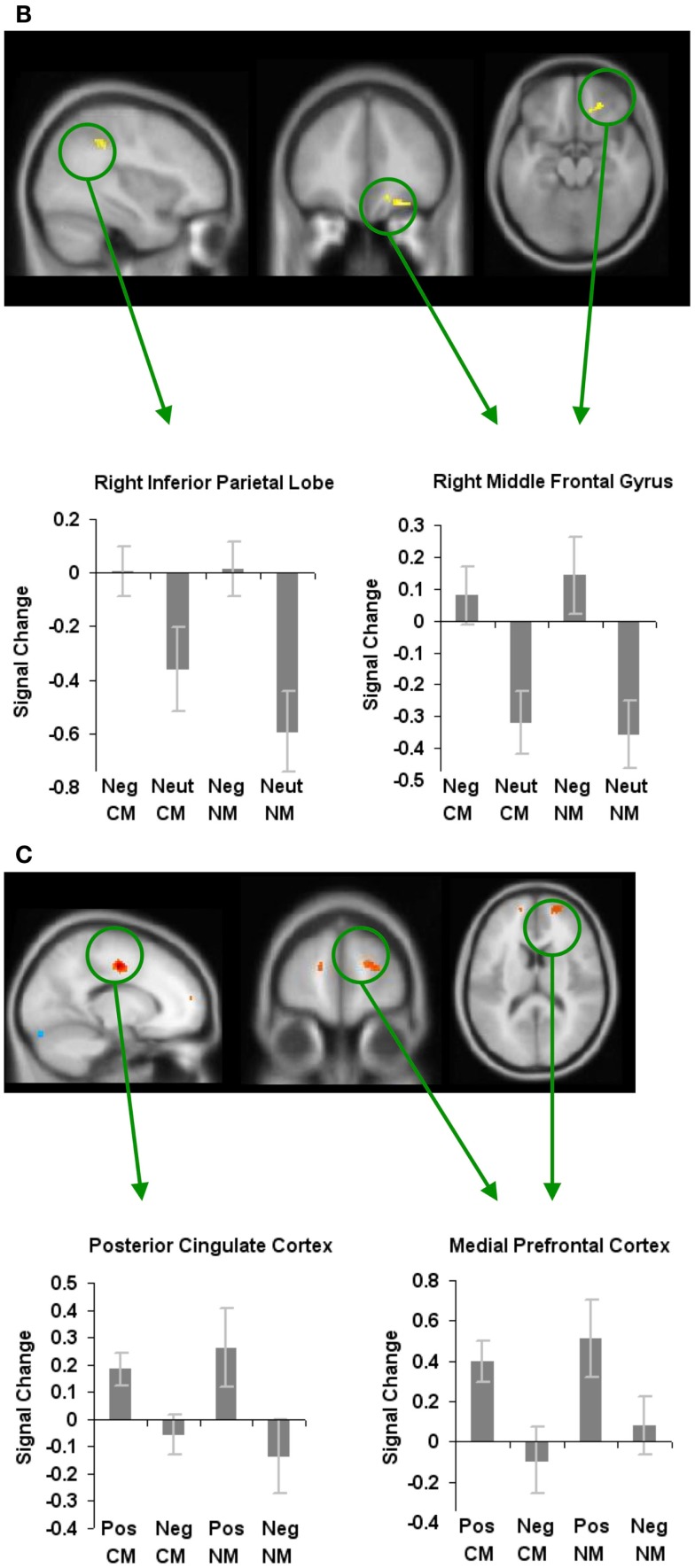
**Whole-brain activation showing signal change for affective stimuli modulating metaphoric processes in general: (A) reveals signal change for positively valenced metaphors versus neutral metaphors, (B) reveals signal change for negatively valenced metaphors versus neutral metaphors, and (C) reveals signal change for positively valenced metaphors versus negatively valenced metaphors**. All clusters are illustrated at a threshold of *p* < 0.001 (uncorrected) combined with a cluster size of at least 50 voxels or 400 mm^3^ in volume. Mean beta weights for each condition extracted from the clusters illustrated in the specific contrasts of interest are revealed in the bar charts.

In order to examine how valence modulates metaphoric processes in general, we first contrasted positively valenced versus neutral metaphors, and found increased activation the mPFC, PCC, and the RIPL. No regions showed deactivation for positively valenced versus neutral metaphors (Figure 3A; Table 1).

Next, when we contrasted negatively valenced versus neutral metaphors, we found increased signal change in two regions: the RIPL and the right middle frontal gyrus (RMFG). There were no regions that showed deactivation for negatively valenced versus neutral metaphors (Figure 3B; Table 1).

Finally, we found that the mPFC and PCC were the only two regions that mediated the processing of positively valenced metaphors to a greater extent when compared to negatively valenced metaphors. We did not find any regions that met our statistical threshold criteria which showed deactivation for positively valenced versus negatively valenced metaphors (Figure 3C; Table 1).

#### Which brain regions show signal change for positively valenced novel metaphors versus neutral novel metaphors (PosNM versus NeutNM), and for positively valenced conventional metaphors versus neutral conventional metaphors (PosCM versus NeutCM)?

**Figure 4:**
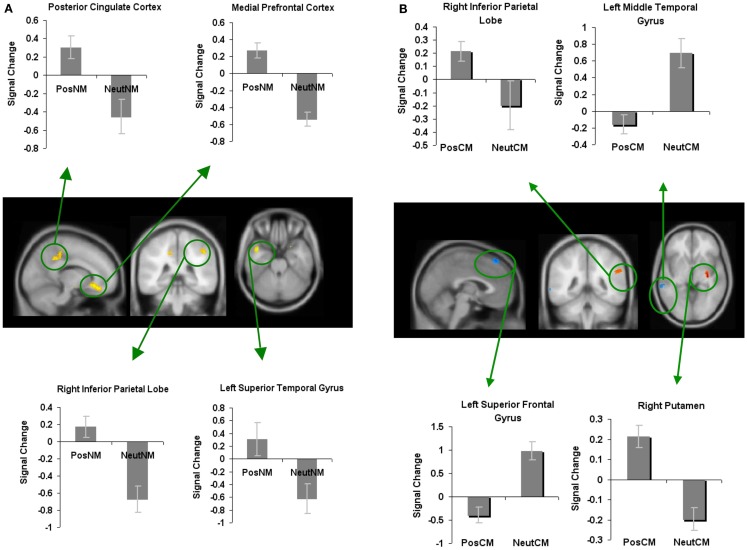
**Whole-brain activation showing signal change for the positively valenced versus neutral metaphoric type-specific contrasts: (A) reveals signal change for positively valenced novel metaphors versus neutral novel metaphors and (B) reveals signal change for positively valenced conventional metaphors versus neutral conventional metaphors**. All clusters are illustrated at a threshold of *p* < 0.001 (uncorrected) combined with a cluster size of at least 50 voxels or 400 mm^3^ in volume. Mean beta weights for each condition extracted from the clusters illustrated in the specific contrasts of interest are revealed in the bar charts.

In order to investigate where in the brain positively valenced stimuli specifically modulated the processing of novel metaphors, we contrasted positively valenced novel metaphors with neutral novel metaphors. We found increased activation in several regions such as the mPFC, the PCC, the LSTG, and the RIPL. There were no regions that showed deactivation for positively valenced novel metaphors versus neutral novel metaphors (Figure 4A; Table 1).

Next, in order to find whether positively valenced stimuli modulated neural signal differently depending on the type of metaphoric process (novel compared to conventional), we contrasted positively valenced conventional metaphors with neutral conventional metaphors (PosCM versus NeutCM). We found increased activation for positively valenced versus neutral conventional metaphors in the putamen and the right supramarginal gyrus/RIPL. We also found deactivation for positively valenced versus neutral conventional metaphors in the left middle temporal gyrus and the left superior frontal gyrus (Figure 4B; Table 1).

#### Which brain regions show signal change for negatively valenced novel metaphors versus neutral novel metaphors (NegNM versus NeutNM), and for negatively valenced conventional metaphors versus neutral conventional metaphors (NegCM versus NeutCM)?

**Figure 5:**
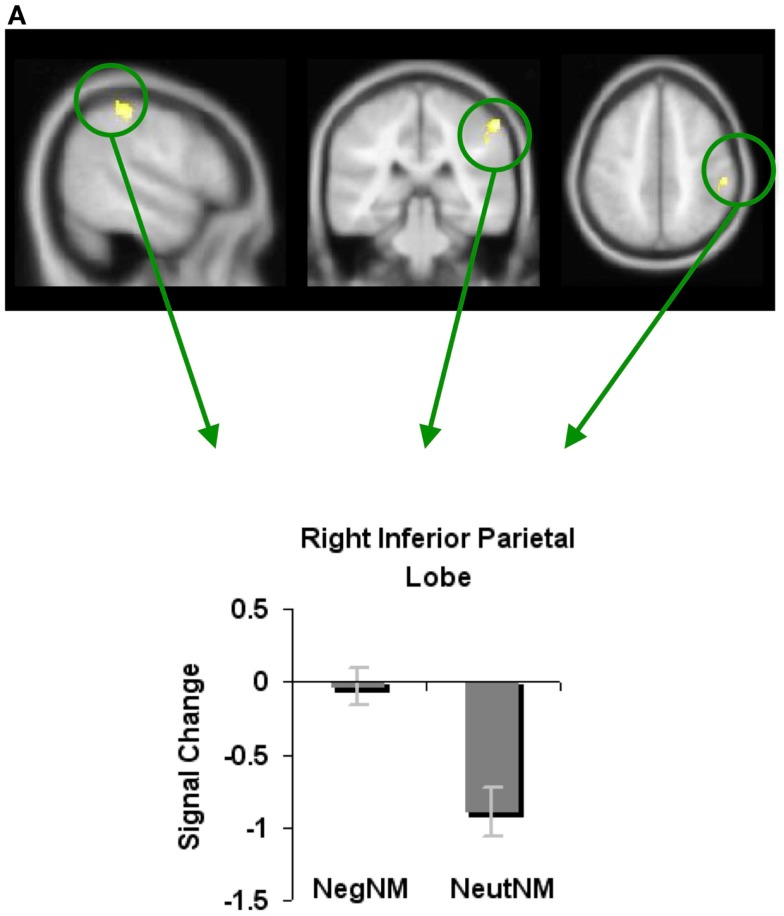

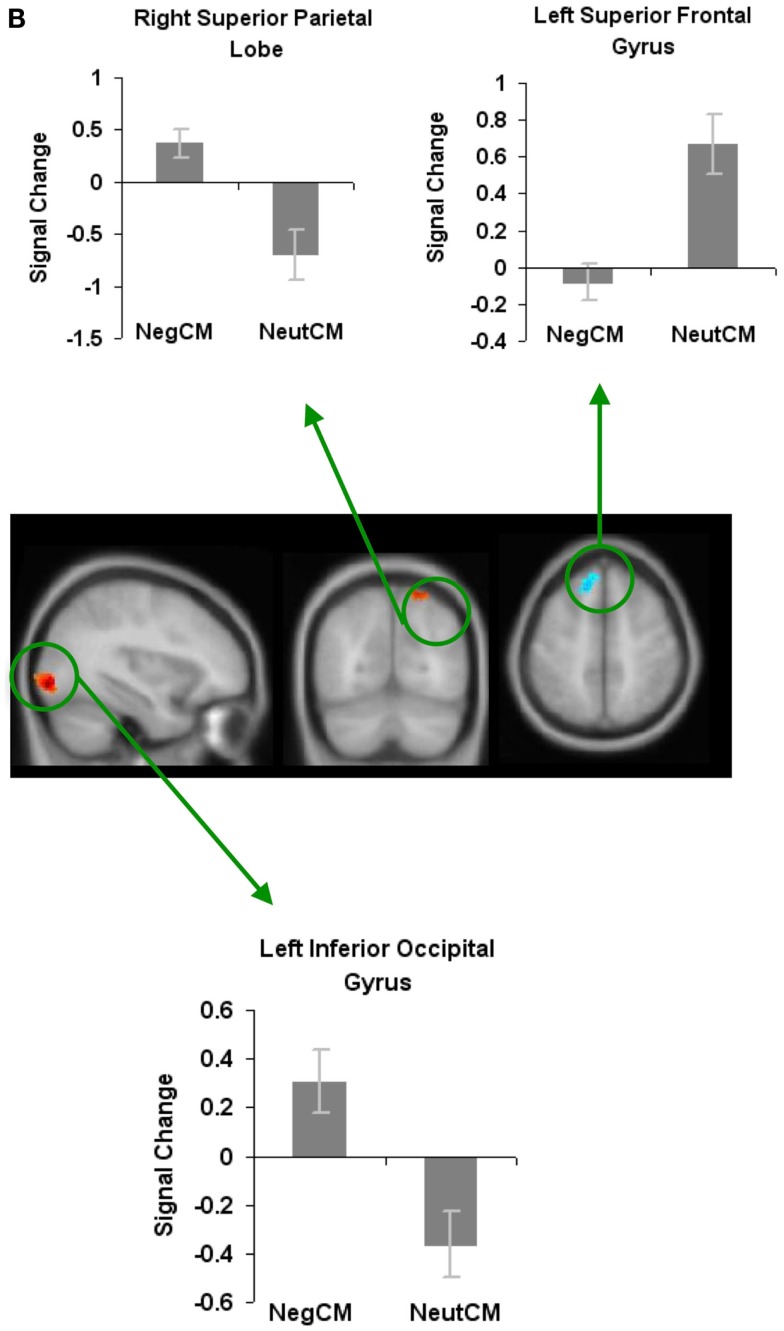
**Whole-brain activation showing signal change for the negatively valenced versus neutral metaphoric type-specific contrasts (A) reveals signal change for negatively valenced novel metaphors versus neutral novel metaphors and (B) reveals signal change for negatively valenced conventional metaphors versus neutral conventional metaphors**. All clusters are illustrated at a threshold of *p* < 0.001 (uncorrected) combined with a cluster size of at least 50 voxels or 400 mm^3^ in volume. Mean beta weights for each condition extracted from the clusters illustrated in the specific contrasts of interest are revealed in the bar charts.

We next examined whether novel metaphoric processes were modulated differently depending on the specific affective process at hand. We, therefore, contrasted neural activity associated with negatively valenced versus neutral novel metaphorical processes (NegNM versus NeutNM), and found increased signal change (i.e., less deactivation) in only one region: the RIPL. No brain areas showed reduced signal change for negatively valenced versus neutral novel metaphors (Figure 5A; Table 1).

When we contrasted negatively valenced conventional metaphors with neutral conventional metaphors, we found increased activation in two regions: the left inferior occipital gyrus and the right superior parietal lobe. Only one region, the left superior frontal gyrus, showed deactivation for negatively valenced versus neutral conventional metaphors (Figure 5B; Table 1).

#### Which brain regions show signal change for positively valenced novel metaphors versus negatively valenced novel metaphors (PosNM versus NegNM), and for positively valenced conventional metaphors versus negatively valenced conventional metaphors (PosCM versus NegCM)?

**Figure 6:**
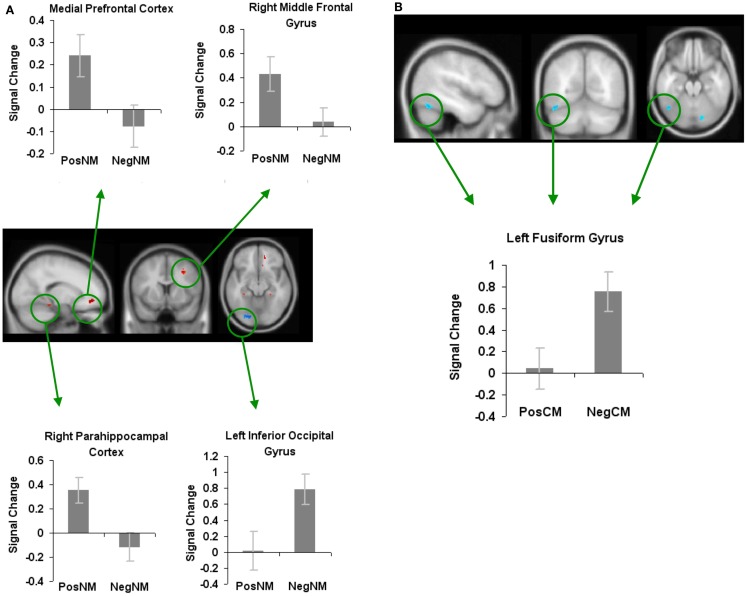
**Whole-brain activation showing signal change for the positively valenced versus negatively valenced metaphoric type-specific contrasts (A) reveals signal change for positively valenced novel metaphors versus negatively valenced novel metaphors (B) reveals signal change for positively valenced conventional metaphors versus negatively valenced conventional metaphors**. All clusters are illustrated at a threshold of *p* < 0.001 (uncorrected) combined with a cluster size of at least 50 voxels or 400 mm^3^ in volume. Mean beta weights for each condition extracted from the clusters illustrated in the specific contrasts of interest are revealed in the bar charts.

In order to find brain areas that were specifically modulated by novel metaphors with positively valenced connotations, we also contrasted positively valenced novel metaphors with negatively valenced novel metaphors (PosNM versus NegNM). We found increased activation in three regions: the mPFC, the right parahippocampal cortex, and the RMFG. We found increased activation for negatively valenced novel metaphors when compared to positively valenced novel metaphors in only one region, the left inferior occipital gyrus (Figure 6A; Table 1).

In keeping with this approach, to find brain areas that were specifically modulated by conventional metaphors with positively valenced connotations, we contrasted positively valenced conventional metaphors with negatively valenced conventional metaphors (PosCM versus NegCM). No regions showed increased activation for PosCM versus NegCM, and only one region, the left fusiform gyrus, showed increased activation for the NegCM versus PosCM contrast (Figure 6B; Table 1).

## Discussion

### Behavioral analyses

When participants viewed positively valenced metaphors (both conventional and novel), they were more accurate and faster at correctly identifying that these metaphors were meaningful when compared to neutral stimuli. The present results are consistent with findings from prior studies which have shown that both positive mood states and positively valenced stimuli are more interconnected compared to neutral states and stimuli (Isen, 1985, 1987a; Ashby et al., 1999; Federmeier et al., 2001; Gasper and Clore, 2002; Bolte et al., 2003; Friedman et al., 2003; Rowe et al., 2007; Subramaniam et al., 2009). Together, our findings suggest that participants were able to form more meaningful judgments for positively valenced stimuli possibly because these stimuli facilitated broader attention/semantic access (Fenske and Eastwood, 2003; Srinivasan and Hanif, 2010).

We did not find any accuracy differences between positively valenced versus negatively valenced metaphors. However, we did find that participants were more accurate at identifying that negatively valenced metaphors were meaningful when compared to neutral metaphors. One possible explanation for this difference is informed by prior research, which has shown that people are more attentive when processing negative stimuli (Hansen and Hansen, 1988; Pratto and John, 1991; Rossell and Nobre, 2004), perhaps due to a primal tendency to direct attention to negative information in adverse situations.

### fMRI analyses

Of all four ROIs that showed repetition enhancement effects, the two-way stimulus type × valence interaction was significant in only one region, the mPFC. Furthermore, planned comparisons revealed that the mPFC was the only region that showed greater activation for positively valenced novel metaphors when compared with negatively valenced novel metaphors and neutral novel metaphors. Together, these data indicate that positively valenced stimuli are likely to enhance metaphoric comprehension, particularly novel metaphoric processes, by regulating attention and cognitive control processes mediated by the mPFC.

In terms of the whole-brain analyses, we found four principal results which were that:

When participants viewed positively valenced metaphors versus neutral metaphors (i.e., for both conventional and novel), they were more accurate and faster at making meaningful judgments via modulation of mPFC, PCC, and RIPL regions. When participants viewed negatively valenced metaphors, they were more accurate, although not faster, at making meaningful judgments when compared to neutral metaphors. Two regions: the RIPL and RMFG were modulated by negatively valenced metaphors. Interestingly, we found that only two regions, the mPFC and PCC, mediated the processing of positively valenced metaphors when compared to negatively valenced metaphors. These two regions were also activated for positively valenced versus neutral metaphors.
Participants identified that positively valenced novel metaphors were meaningful via modulation of the mPFC, PCC, LSTG, and RIPL. Additionally, participants identified that positively valenced conventional metaphors were meaningful through modulation of the RIPL and putamen.
Participants showed increased activation in only one region, the RIPL, when identifying the meaningfulness of negatively valenced novel metaphors compared to neutral novel metaphors. With regard to negatively valenced conventional versus neutral conventional metaphors, participants revealed increased activation within two regions, the left inferior occipital gyrus and the RSPL, known to mediate visual attention processes.
Only one region, the mPFC, showed increased activation for both positively valenced versus neutral and positively valenced versus negatively valenced novel metaphoric processes. Together, these results indicate that the mPFC region specifically mediates the processing of novel unfamiliar stimuli particularly when they are positively valenced.

#### Where and how in the brain does affective valence modulate metaphoric processes in general?

When participants viewed positively valenced metaphors in general (both conventional and novel) when compared to neutral metaphors, they showed increased activation in regions such as the mPFC, PCC, and the RIPL (Figure 3A). These results indicate that positively valenced stimuli facilitate metaphoric comprehension in general via modulation of attention and cognitive control processes supported by the mPFC, PCC, and the RIPL. Greater attention/control may be exerted through a combination of ways, including: facilitating a broadening of attention (Isen, 1985, 1987a), facilitating coarse semantic coding in the RH to access multiple interpretations (Beeman, 1998; Jung-Beeman, 2005; Subramaniam et al., 2012) and facilitating switching processes to select the correct meaningful interpretation (Ashby et al., 1999; Dreisbach and Goschke, 2004; Baumann and Kuhl, 2005). Together, these processes allow easier access to distant semantic associations that connect the word pairs, enabling participants to switch between attentional modes (broad versus focused), to reject interpretations that are meaningless in order to hone in on selecting a sensible interpretation for the novel metaphors.

When we contrasted negatively valenced versus neutral metaphors, we found increased signal change in two regions: the RIPL and the RMFG. It is important to note that signal change in these regions (more in the RIPL) was specifically driven by greater deactivation to the neutral stimuli rather than by increased activation to the negative stimuli (Figure 3B). Neutral stimuli may reduce attention/semantic integrative processes to a greater extent than negative stimuli by deactivating RIPL and RMFG, perhaps because participants considered these stimuli to be less interesting and less demanding of attention.

Finally, we found that the mPFC and PCC were the only two regions that mediated the processing of positively valenced metaphors to a greater extent when compared to negatively valenced metaphors. Both these regions showed the highest level of activation when participants viewed positively valenced novel metaphorical processes specifically, and then when participants also viewed positively valenced conventional metaphors (Figure 3C). Together, these results indicate that positively valenced stimuli may enhance creative metaphoric processes by regulating attention and cognitive control processes mediated by the mPFC/ACC and PCC (Bush et al., 2000; Small et al., 2003; Botvinick et al., 2004; Kerns et al., 2004). Greater control allows for better access and detection of more semantic associations so that participants are better able to formulate or retrieve meaning for the word pairs.

#### How do positively valenced stimuli specifically enhance novel metaphoric comprehension, and conventional metaphoric recall in the brain?

When we contrasted positively valenced with neutral novel metaphors, we found that participants activated a network of regions including the mPFC, PCC, RIPL, and LSTG. These regions support the affective-cognitive interactions required to predispose and facilitate novel metaphoric comprehension. The present findings are in accordance with our previous study, in which we revealed that positive mood states facilitated people’s ability to solve novel creative problems via modulation of the mPFC/ACC (Subramaniam et al., 2009). Novel metaphoric comprehension is also considered a type of creative cognition because it requires that participants are able to recognize distantly related word pairs as meaningful, which they have previously not seen/used together (i.e., unfenced idea). Extending upon our prior findings, in the current study we reveal that positively valenced stimuli can also predispose and facilitate creative novel metaphoric processes by enhancing attention/control processes via mPFC/rostral ACC modulation.

In another previous study, we revealed that when participants computed novel metaphoric expressions, they required greater attention and cognitive control processes associated with the formulation of more closely connected meanings, mediated by regions such as the mPFC, PCC, RIPL, and LSTG (Subramaniam et al., 2012). In the present study, we also extend upon these prior findings by revealing that positively valenced stimuli can facilitate creative novel metaphoric processes by enhancing activity within this specific network of neural regions. We know from prior research that the rostral ACC is implicated in attention and cognitive control processes in the frontal cortex (Bush et al., 2000; Botvinick et al., 2004; Kerns et al., 2004) and the PCC is thought to mediate visuospatial attention processes in the posterior cortex (Small et al., 2003). The present findings, therefore, suggest that positively valenced stimuli may facilitate novel metaphoric comprehension by enhancing attention/control processes within the mPFC and PCC. Greater control promotes greater detection of a broader range of associations that may link the word pairs together in order for participants to select a meaningful interpretation for the unfamiliar word pairs.

Additionally, prior research has revealed that the RH is activated when participants process distantly related meanings and performs more “coarse semantic coding” (Jung-Beeman, 2005). As such, the present findings suggest that positively valenced stimuli may also facilitate this process of formulating meaning from remotely connected novel metaphors by enhancing activation of the RIPL. We also found increased activation in the LSTG during positive versus neutral novel metaphoric comprehension. This result is consistent with the theory that the LH performs more fine semantic coding (Jung-Beeman, 2005) during the integration and selection of a more closely connected meaning (Geschwind, 1965; Binder et al., 2009; Bambini et al., 2011). Together, these findings reveal that positively valenced stimuli may enhance creative novel metaphoric processes partly by modulating the RH initially during more coarse semantic coding required for the integration of distant semantic associations. Positively valenced stimuli are also likely to promote attention/cognitive control processes supported by the mPFC/PCC, so that participants are subsequently better able to select more closely connected salient meanings via fine semantic coding supported by the LSTG.

We also found that positively valenced stimuli did modulate neural signal differently depending on the type of metaphor that the participants had viewed. This was an expected finding given that participants engaged in different processes when viewing the two types of metaphors. Participants were building a meaning when they viewed novel metaphors whereas when they viewed conventional metaphors, they were recalling a meaning. When we contrasted positively valenced conventional metaphors with neutral conventional metaphors, we found increased activation in two regions: the RIPL, and the putamen. As noted earlier, there is a plethora of evidence that suggests that both positive mood states and positively valenced stimuli give rise to broader cognitive elaboration, interconnectedness, and complexity (Isen, 1985, 1987a; Ashby et al., 1999; Federmeier et al., 2001; Gasper and Clore, 2002; Bolte et al., 2003; Friedman et al., 2003; Rowe et al., 2007; Subramaniam et al., 2009). The right temporal/parietal cortex is cytoarchitectonically suited to mediate the recall of distant semantic associations via coarse semantic coding (Hutsler and Galuske, 2003; Jung-Beeman, 2005) with wider and more distal dendritic branches. Thus, it represents a reasonable candidate for modulation by positively valenced conventional stimuli to promote a broader recall of familiar associations.

The putamen is also known to mediate positive-valenced stimuli, and supports both positive mood and reward processing (Elliott et al., 2000; McClure et al., 2003; Tobler et al., 2006). Therefore, it is not surprising that when participants recalled positively valenced conventional metaphors, they recruited the putamen to a greater extent when compared to neutral conventional metaphors.

#### Where and how in the brain do negatively valenced stimuli modulate novel metaphoric comprehension, and conventional metaphoric recall?

When we contrasted negatively valenced novel metaphors with neutral novel metaphors, we found that participants showed relatively increased signal change in the RIPL. As noted earlier, the right temporal/parietal cortex is better suited to mediate the integration of distant semantic associations associated with positively valenced but not negatively valenced stimuli (Isen, 1985, 1987a). Therefore, it may be surprising as to why the RIPL was recruited for negatively valenced stimuli. Further analyses revealed that participants, in fact, showed deactivation (rather than activation) in RIPL to negatively valenced stimuli relative to baseline. This result indicates that negatively valenced stimuli may reduce distant semantic integrative processes by deactivating RIPL, albeit to a lesser extent when compared to neutral stimuli.

When we compared negatively valenced conventional metaphors with neutral conventional metaphors, we found increased activation in two regions: the RSPL and the left inferior occipital gyrus. These two regions are implicated in visual attention processes, consistent with prior studies which have shown that participants are more vigilant when processing negative stimuli (Hansen and Hansen, 1988; Pratto and John, 1991; Rossell and Nobre, 2004).

#### Do positively valenced versus negatively valenced stimuli enhance novel metaphoric comprehension by enhancing mPFC activation?

Next, we contrasted positively valenced versus negatively valenced novel metaphors. Our goal was to examine whether regions such as the mPFC that revealed increased activation for positively valenced versus neutral novel metaphors, also showed increased activation for positively valenced versus negatively valenced novel metaphoric processes. This result would indicate that mPFC modulation would be specific to positively valenced novel metaphors, rather than due to non-specific effects of valenced stimuli (i.e., due to both positive and negative valence). We found three regions including the mPFC, RMFG, and right parahippocampal cortex, which all showed greater activation for positively valenced versus negatively valenced novel metaphoric processes. Together, these results suggest that positively valenced stimuli modulated both the mPFC and RMFG implicated in attention and cognitive control processes, which are critical for building novel metaphoric meanings. Only the mPFC region revealed activation for positively valenced novel metaphors when compared to both neutral and negatively valenced metaphors, indicating regional specific effects of positive stimuli. Thus, mPFC modulation during novel metaphoric comprehension, was specific to stimuli that were positively valenced. We also found that positively valenced stimuli modulated the formation and storage of these creative novel metaphoric meanings into long-term memory to a greater extent than negatively valenced stimuli via activation of the right parahippocampal cortex. Only one region, the left inferior occipital gyrus, implicated in visual perception showed increased activation for negatively valenced novel metaphors versus positively valenced novel metaphors.

We did not find any regions that showed greater activation for positively valenced conventional metaphors versus negatively valenced conventional metaphors when participants were recalling a familiar meaning. However, we did find one region, the left fusiform gyrus, which showed greater activation for negatively valenced conventional metaphors versus positively valenced conventional metaphors. This result indicates that when participants recalled a conventional meaning, they directed greater visual attention to negative information via modulation of the fusiform gyrus.

### Limitations

The present findings reveal that a positive valence can enhance metaphorical comprehension, specifically creative novel metaphoric comprehension. Yet, one limitation of the current study is the modest sample size. Limited sample power precluded FDR correction for multiple comparisons at a whole-brain level. Therefore, the whole-brain analyses must be interpreted with caution. However, the convergent findings from the ROI analyses as well as the four principal whole-brain analyses all reveal that participants correctly made more meaningful judgments when metaphors were positively valenced via modulation of the mPFC. The mPFC is a region that mediates attention processes, enabling participants to have a greater scope of attention in order to detect many different possible associations that may link the unfamiliar word pairs together. Increased control mediated by the mPFC, also promotes the elimination of interpretations that are meaningless in order for participants to select a reasonable interpretation that links the novel words together in a meaningful way. With the convergent findings from the ROI analyses and the four principal whole-brain fMRI analyses which all reveal that the mPFC mediates metaphoric processes, particularly creative novel metaphoric processes, that were specifically facilitated by positively valenced stimuli but not by negatively valenced stimuli, we believe that the current results will be replicated with a larger sample size.

## Conclusion

In summary, the combined use of ROI and whole-brain analyses illustrate that participants were more accurate and faster when processing the meaning for metaphors that were positively valenced, which was accompanied by enhanced neural signal in the mPFC. The mPFC, more consistently than the PCC, was activated during positively valenced metaphors in general, but specifically during positively valenced novel metaphors. The present findings reveal that mPFC modulation of metaphoric processes, particularly novel metaphoric processes, was specific to positively] valenced stimuli but not to negatively valenced stimuli. Together, these results reveal that affective valence, specifically a positive valence, can modulate mPFC activation, likely through enhancing attention and cognitive control processes, to predispose and facilitate the more creative aspects of semantic processing, i.e., the comprehension of novel metaphoric expressions. These findings suggest that behavioral treatments which increase hedonic capacity and/or harness hedonic mechanisms in the brain may help to generate improved creative cognitive performance. A better understanding of the neural mechanisms that mediate emotion-cognition interactions in creative language and cognition is warranted in the future.

## Conflict of Interest Statement

The authors declare that the research was conducted in the absence of any commercial or financial relationships that could be construed as a potential conflict of interest.

## Appendix

**Table A1:** **Examples of conventional metaphors, novel metaphors, and unrelated word-pairs**.

Conventional metaphors	Novel metaphors	Unrelated word pairs
Sweet revenge	Flimsy spirit	Cheek brains
Biting remark	Tidy prose	Splendor dog
Nature call	Sunny enthusiasm	Teeth tree
Lion heart	Imagination flight	Opinion vegetable
Dark thoughts	Realization spark	Vision ticket
Face value	Romantic dexterity	Stocks hand
Weighty decision	Combustible look	Juicy iron
Absolute pitch	Mercy blanket	Grain computer
Glowing review	Concrete inhibitions	Road bees
Silken lies	Blue moaning	Withered depth

## References

[B1] Amanzio M. Geminiani G. Leotta D. Cappa S. (2008). Metaphor comprehension in Alzheimer’s disease: novelty matters. Brain Lang. 107, 1–10 10.1016/j.bandl.2007.08.003 17897706

[B2] Ashby F. G. Isen A. M. Turken U. (1999). A neuropsychological theory of positive affect and its influence on cognition. Psychol. Rev. 106, 529–550 10.1037/0033-295X.106.3.529 10467897

[B3] Bambini V. Gentili C. Ricciardi E. Bertinetto P. M. Pietrini P. (2011). Decomposing metaphor processing at the cognitive and neural level through functional magnetic resonance imaging. Brain Res. Bull. 86, 203–216 10.1016/j.brainresbull.2011.07.015 21803125

[B4] Baumann N. Kuhl J. (2005). Positive affect and flexibility: overcoming the precedence of global over local processing of visual information. Motiv. Emot. 29, 123–134 10.1007/s11031-005-7957-1

[B5] Beeman M. (1998). “Coarse semantic coding and discourse processing,” in Right Hemisphere Language Processing: Perspectives from Cognitive Neuroscience, eds Beeman M. Chiarello C. (Mahwah: Erlbaum), 255–284

[B6] Binder J. R. Desai R. H. Graves W. W. Conant L. L. (2009). Where is the semantic system? A critical review and meta-analysis of 120 functional neuroimaging studies. Cereb. Cortex 19, 2767–2796 10.1093/cercor/bhp055 19329570 PMC2774390

[B7] Bolte A. Goschke T. Kuhl J. (2003). Effects of positive and negative mood on implicit judgments of semantic coherence. Emot. Intuition 14, 416–421

[B8] Botvinick M. M. Cohen J. D. Carter C. S. (2004). Conflict monitoring and anterior cingulate cortex: an update. Trends Cogn. Sci. (Regul. Ed.) 8, 539–546 10.1016/j.tics.2004.10.003 15556023

[B9] Brownell H. H. Potter H. H. Michelow D. Gardner H. (1984). Sensitivity to lexical denotation and connotation in brain-damaged patients: a double dissociation? Brain Lang. 22, 253–265 10.1016/0093-934X(84)90093-2 6204711

[B10] Bush G. Luu P. Posner M. I. (2000). Cognitive and emotional influences in anterior cingulate cortex. Trends Cogn. Sci. (Regul. Ed.) 4, 215–222 10.1016/S1364-6613(00)01483-2 10827444

[B11] Chiappe D. L. Chiappe P. (2007). The role of working memory in metaphor production and comprehension. J. Mem. Lang. 56, 172–188 10.1016/j.jml.2006.11.006

[B12] Dreisbach G. Goschke T. (2004). How positive affect modulates cognitive control: reduced perseveration at the cost of increased distractibility. J. Exp. Psychol. Learn Mem. Cogn. 30, 343–353 10.1037/0278-7393.30.2.343 14979809

[B13] Elliott R. Friston K. J. Dolan R. J. (2000). Dissociable neural responses in human reward systems. J. Neurosci. 20, 6159–6165 10934265 10.1523/JNEUROSCI.20-16-06159.2000 PMC6772605

[B14] Estrada C. A. Young M. Isen A. M. (1994). Positive affect influences creative problems solving and reported source of practice satisfaction in physicians. Motiv. Emot. 18, 285–299 10.1007/BF02856470

[B15] Faust M. Mashal N. (2007). RH advantage in processing novel metaphoric expressions: a DVF Study. Neuropsychologia 45, 860–870 10.1016/j.neuropsychologia.2006.08.010 17010392

[B16] Federmeier K. D. Kirson D. A. Moreno E. M. Kutas M. (2001). Effects of transient, mild mood states on semantic memory organization and use: an event-related potential investigation in humans. Neurosci. Lett. 305, 149–152 10.1016/S0304-3940(01)01843-2 11403927

[B17] Fenske M. J. Eastwood J. D. (2003). Modulation of focused attention by faces expressing emotion: evidence from Flanker tasks. Emotion 3, 327–343 10.1037/1528-3542.3.4.327 14674827

[B18] Fraser B. (1998). “The interpretation of novel metaphors,” in Metaphor and Thought, ed. Ortony A. (New York, NY: Cambridge University Press), 329–341

[B19] Friedman R. Fishbein A. Förster J. Werth L. (2003). Attentional priming effects on creativity. Creat. Res. J. 15, 277–286 10.1080/10400419.2003.9651420

[B20] Gasper K. Clore G. L. (2002). Attending to the big picture: mood and global versus local processing of visual information. Psychol. Sci. 13, 34–40 10.1111/1467-9280.00406 11892776

[B21] Gentner D. (1983). Structure-mapping: a theoretical framework for analogy. Cogn. Sci. 7, 155–170 10.1207/s15516709cog0702_3

[B22] Geschwind N. (1965). Disconnexion syndromes in animals and man II. Brain 88, 585–644 10.1093/brain/88.3.585 5318824

[B23] Gibbs R. W. (1980). Spilling the bean on understanding and memory of idioms in conversation. Mem. Cogn. 8, 449–456 10.3758/BF03213418 7382816

[B24] Gibbs R. W. (1994). The Poetics of Mind. Cambridge: Cambridge University Press

[B25] Giora R. (1997). Understanding figurative and literal language: the graded salience hypothesis. Cogn. Linguist. 7, 183–206 10.1515/cogl.1997.8.3.183

[B26] Giora R. (2003). On Our Mind: Salience, Context and Figurative Language. New York: Oxford University Press

[B27] Giora R. (2007). Is metaphor special? Brain Lang. 100, 111–114 10.1016/j.bandl.2006.08.001 16956657

[B28] Giora R. Fein O. (1999a). Irony: context and salience. Metaphor Symbol 14, 241–257 10.1207/S15327868MS1404_1

[B29] Giora R. Fein O. (1999b). On understanding familiar and less familiar figurative language. J. Pragmat. 31, 1601–1618 10.1016/S0378-2166(99)00002-8

[B30] Glucksberg S. (2003). The psycholinguistics of metaphor. Trends Cogn. Sci. (Regul. Ed.) 7, 92–96 10.1016/S1364-6613(02)00040-2 12584028

[B31] Grice H. P. (1975). “Logic and conversation,” in Speech Acts: Syntax and Semantics, Vol. 3, eds Cole P. Morgan J. (New York: Academic Press), 41–58

[B32] Hansen R. D. Hansen C. H. (1988). Repression of emotionally tagged memories: the architecture of less complex emotions. J. Pers. Soc. Psychol. 55, 811–818 10.1037/0022-3514.55.5.811 3210148

[B33] Hutsler J. Galuske R. A. (2003). Hemispheric asymmetries in cerebral cortical networks. Trends Neurosci. 26, 429–435 10.1016/S0166-2236(03)00198-X 12900174

[B34] Isen A. M. (1985). Asymmetry of happiness and sadness in effects on memory in normal college students: comment on Hasher, Rose, Zackes, Sanft, and Doren. J. Exp. Psychol. Gend. 114, 388–391 10.1037/0096-3445.114.3.388

[B35] Isen A. M. (1987a). “Positive affect, cognitive processes, and social behavior,” in Advances in Experimental Social Psychology, ed. Berkowitz L. (San Diego, CA: Academic Press), 203–253

[B36] Isen A. M. (1999). “On the relationship between affect and creative problem solving,” in Affect, Creative Experience and Psychological Adjustment, ed. Russ S. W. (Philadelphia, PA: Brunner/Mazel), 3–17

[B37] Isen A. M. Daubman K. A. Nowicki P. (1987b). Positive affect facilitates creative problem solving. J. Pers. Soc. Psychol. 52, 1122–1131 10.1037/0022-3514.52.6.1122 3598858

[B38] Jung-Beeman M. (2005). Bilateral brain processes for comprehending natural language. Trends Cogn. Sci. (Regul. Ed.) 9, 512–518 10.1016/j.tics.2005.09.009 16214387

[B39] Kaufmann G. Vosburg S. K. (1997). “Paradoxical” mood effects on creative problem-solving. Cogn. Emot. 11, 151–170 10.1080/026999397379971

[B40] Kerns J. G. Cohen J. D. MacDonald A. W. III Cho R. Y. Stenger V. A. Carter C. S. (2004). Anterior cingulate conflict monitoring and adjustments in control. Science 303, 1023–1026 10.1126/science.1089910 14963333

[B41] Kucera H. Francis W. N. (1967). Computational Analysis of Present Day American English. Providence: Brown University Press

[B42] Mashal N. (in press). The role of working memory in the comprehension of unfamiliar and familiar metaphors. Lang. Cogn.

[B43] Mashal N. Faust M. (2009). Conventionalization of novel metaphors: a shift in hemispheric asymmetry. Laterality 14, 573–589 19253086 10.1080/13576500902734645

[B44] Mashal N. Faust M. Hendler T. (2005). The role of the right hemisphere in processing nonsalient metaphorical meanings: application of principal components analysis to fMRI data. Neuropsychologia 43, 2084–2100 10.1016/j.neuropsychologia.2005.03.019 16243053

[B45] Mashal N. Faust M. Hendler T. Jung-Beeman M. (2007). An fMRI investigation of the neural correlates underlying the processing of novel metaphoric expressions. Brain Lang. 100, 115–126 10.1016/j.bandl.2005.10.005 16290261

[B46] McClure S. M. Berns G. S. Montague P. R. (2003). Temporal prediction errors in a passive learning task activate human striatum. Neuron 38, 339–346 10.1016/S0896-6273(03)00154-5 12718866

[B47] Ogawa S. Tank D. W. Menon R. Ellermann J. M. Kim S. G. Merkle H. (1992). Intrinsic signal changes accompanying sensory stimulation: functional brain mapping with magnetic resonance imaging. Proc. Natl. Acad. Sci. U.S.A. 89, 5951–5955 10.1073/pnas.89.18.8557 1631079 PMC402116

[B48] Pobric G. Mashal N. Faust M. Lavidor M. (2008). The causal role of the right cerebral hemisphere in processing novel metaphoric expressions taken from poetry: a TMS study. J. Cogn. Neurosci. 20, 170–181 10.1162/jocn.2008.20005 17919080

[B49] Pratto F. John O. P. (1991). Automatic vigilance: the attention-grabbing power of negative social information. J. Pers. Soc. Psychol. 61, 380–391 10.1037/0022-3514.61.3.380 1941510

[B50] Rossell S. L. Nobre A. C. (2004). Semantic priming of different affective categories. Emotion 4, 354–363 10.1037/1528-3542.4.4.354 15571434

[B51] Rowe G. Hirsch J. B. Anderson A. K. (2007). Positive affect increases the breadth of attentional selection. Proc Natl. Acad. Sci. U.S.A. 101, 383–388 10.1073/pnas.0605198104 17182749 PMC1765470

[B52] Sass K. Habel U. Sachs O. Huber W. Gauggel S. Kircher T. (2012). The influence of emotional associations on the neural correlates of semantic priming. Hum. Brain Mapp. 33, 676–694 10.1002/hbm.21241 21520342 PMC6870432

[B53] Small D. M. Gitelman D. R. Gregory M. D. Nobre A. C. Parrish T. B. Mesulam M. M. (2003). The posterior cingulate and medial prefrontal cortex mediate the anticipatory allocation of spatial attention. Neuroimage 18, 633–641 10.1016/S1053-8119(02)00012-5 12667840

[B54] Srinivasan N. Hanif A. (2010). Global-happy and local-sad: perceptual processing affects emotion identification. Cogn. Emot. 24, 1062–1069 10.1080/02699930903101103

[B55] Subramaniam K. Faust M. Beeman M. Mashal N. (2012). The repetition paradigm: enhancement of novel metaphors and suppression of conventional metaphors in the left inferior parietal lobe. Neuropsychologia 50, 2705–2719 10.1016/j.neuropsychologia.2012.07.020 22867993

[B56] Subramaniam K. Kounios J. Parrish T. B. Jung-Beeman M. (2009). A brain mechanism for facilitation of insight by positive affect. J. Cogn. Neurosci. 21, 415–432 10.1162/jocn.2009.21057 18578603

[B57] Tobler P. N. O’Doherty P. Dolan R. J. Schultz W. (2006). Human neural learning depends on reward prediction errors in the blocking paradigm. J. Neurophysiol. 95, 301–310 10.1152/jn.00762.2005 16192329 PMC2637603

